# A New Edible Film to Produce In Vitro Meat

**DOI:** 10.3390/foods9020185

**Published:** 2020-02-13

**Authors:** Nicole Orellana, Elizabeth Sánchez, Diego Benavente, Pablo Prieto, Javier Enrione, Cristian A. Acevedo

**Affiliations:** 1 Centro de Biotecnología, Universidad Técnica Federico Santa María, Avenida España 1680, Valparaíso 2340000, Chile; nicole.orellana@usm.cl (N.O.); elizabeth.sanchez@usm.cl (E.S.); 2 Departamento de Ingeniería en Diseño, Universidad Técnica Federico Santa María, Avenida España 1680, Valparaíso 2340000, Chile; diego.benavente@alumnos.usm.cl (D.B.); pablo.prieto@usm.cl (P.P.); 3 Biopolymer Research and Engineering Lab, Facultad de Medicina, Universidad de Los Andes, Monseñor Álvaro del Portillo 12455, Las Condes, Santiago 7550000, Chile; jenrione@uandes.cl; 4 Departamento de Física, Universidad Técnica Federico Santa María, Avenida España 1680, Valparaíso 2340000, Chile

**Keywords:** cultured meat, edible film, in vitro meat, micromolding, muscle cells

## Abstract

In vitro meat is a novel concept of food science and biotechnology. Methods to produce in vitro meat employ muscle cells cultivated on a scaffold in a serum-free medium using a bioreactor. The microstructure of the scaffold is a key factor, because muscle cells must be oriented to generate parallel alignments of fibers. This work aimed to develop a new scaffold (microstructured film) to grow muscle fibers. The microstructured edible films were made using micromolding technology. A micromold was tailor-made using a laser cutting machine to obtain parallel fibers with a diameter in the range of 70–90 µm. Edible films were made by means of solvent casting using non-mammalian biopolymers. Myoblasts were cultured on flat and microstructured films at three cell densities. Cells on the microstructured films grew with a muscle fiber morphology, but in the case of using the flat film, they only produced unorganized cell proliferation. Myogenic markers were assessed using quantitative polymerase chain reaction. After 14 days, the expression of desmin, myogenin, and myosin heavy chain were significantly higher in microstructured films compared to the flat films. The formation of fiber morphology and the high expression of myogenic markers indicated that a microstructured edible film can be used for the production of in vitro meat.

## 1. Introduction

In vitro or cultured meat has emerged as a novel concept in the field of food science and technology, which requires novel biotechnological tools in order to be investigated and developed. In vitro meat is real meat produced by in vitro culture of cells through biotechnology tools, avoiding the slaughter of farmed animals [1,2,3,4,5,6]. An historic date was 5 August 2013, when Professor Mark Post showed to the world a hamburger made of in vitro meat. From that day, the scientific development of in vitro meat has been growing.

The benefits of industrialization of in vitro meat are related to animal welfare, food hazards, human health, and environmental impact [1,2,3,4,5,6]. Three motivators have been identified to investigate the production of livestock meat alternatives: (a) with the predicted substantial increase in meat demand, we will quickly run out of production capacity, as already a large proportion of arable land is dedicated to livestock feeding and management; b) there is a growing concern about the environmental impact of livestock breeding; and (c) high density herding and slaughtering has sparked societal concerns about animal welfare and public health [1].

Currently, the production of in vitro meat in large quantities is not economically viable, as the knowledge about muscle tissue engineering was generated in the field of medical applications. However, more basic research regarding optimization and production of muscle tissue for food products is necessary [7]. To make in vitro meat an economically viable food, it is necessary to investigate three key components, which are animal-free media, scaffolds [8], and bioreactors [9]. That is, methods to produce in vitro meat commonly employ the growth of myoblasts on a scaffold suspended in a serum-free culture medium in a bioreactor [3]. A scaffold is a matrix (soft material) where the anchorage-dependent cells (e.g., muscle cells) can adhere, remain viable, proliferate, and differentiate [4,8]. In addition, the use of mammalian components in the scaffold should be avoided to effectively reduce the slaughter of bovines [2].

Muscle cells, such as other adherent cell types, can be proliferated and differentiated when they are cultivated in scaffolds. Myoblasts have been shown to remain viable when cultured on non-edible commercial scaffolds such as Matrigel [10], or edible mammalian materials such as porcine gelatin [11] or bovine fibrin [7]. However, to the authors’ knowledge, there are no available commercial scaffolds based on non-mammalian compounds for in vitro meat currently in the market. The scientific research on scaffolds for in vitro meat are still scarce, and they are focused on the development of microstructures to align muscle tissue formation [2,11].

In a prior work, we developed an optimized formulation to make edible scaffolds suitable for myoblast culture based on non-mammalian components [8]. The scaffold was formulated with a mix of three marine biopolymers (salmon gelatin, alginate, and agarose), where salmon gelatin performs as functional macromolecule containing RGD-sequences that promote cell adhesion and proliferation [12,13,14], alginate as crosslinker in contact with calcium, and agarose as gelling agent [8]. This edible scaffold was fabricated by freeze-drying, obtaining a microporous material where the myoblasts grew adequately, but the differentiation from myoblasts (muscle progenitor cell) to myocytes and finally muscle fiber were not researched.

The microstructure is a key factor in the design of scaffolds (films) for muscle cell proliferation and differentiation; the morphology should be oriented to generate alignments for the myogenic processes to take place [15]. Surface modifications by using oriented collagen fibers [16], oriented pore [15] electrospinning [17], and fibrous gelatin [11] have been used with good results in the field of muscle tissue engineering. It has been studied that the culture of myoblast on parallel microgrooves can also guide the muscle tissue formation [2,18].

In our previous research, we proved that surface modification engraving microchannels induce alignment of the cells as a muscle-fiber [2]. This allowed us to culture muscle cells with fiber-like morphology and to express early biological markers as desmin and myogenin. Nevertheless, the expression of late marker of myogenesis as myosin heavy chain was not investigated. Myosin heavy chain is widely accepted as a biomarker of final myogenic differentiation [19,20,21]. We strongly believe that the use of high-resolution technology to engrave tailor-made microchannels improves the formation of muscle fiber onto edible materials, allowing the expression of myosin heavy chain.

This work aimed to develop a new scaffold (microstructured film) using micromolding technology to grow functional muscle fibers in the context of in vitro meat production.

## 2. Materials and Methods

### 2.1. Microstructured Mold Fabrication

**Figure 1:**
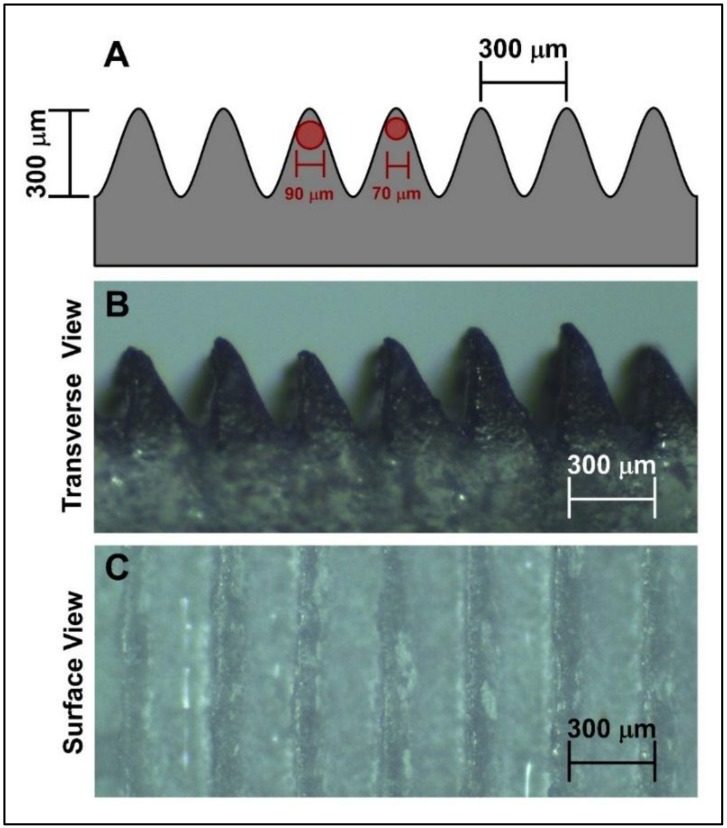
Design of the microstructured mold. (**A**) Schematic representation of the mold (red: theoretical fiber sizes). (**B**) Image of the acrylic mold using stereoscopic microscope (transverse view). (**C**) Image of the acrylic mold using stereoscopic microscope (surface view).

The microstructured mold featuring microchannels was fabricated using acrylic material. For this, an acrylic plate was engraved using a laser cutting machine (Acctek, model AKJ1390, Jinan, China) to obtain parallel microchannels. The theoretical tailor-made design of the microchannels is shown in Figure 1A. A symmetric design of the mold was performed to obtain an analogous shape on the film. The designed mold aimed to obtain microchannels where a muscle fiber of 70–90 µm diameter potentially could grow (see Figure 1A). This theoretical size is in the range of reported fiber for various meat types such as beef (≈70 µm) and pork (≈90 µm) [22].

The manufactured mold is shown in Figure 1B,C. The microchannel width and height were both 300 µm. This microstructure was very similar to the proposed theoretical design and was adequate enough to grow parallel muscle fiber.

### 2.2. Edible Film Preparation

The edible films were prepared by cold-casting using micromolding. In addition, a flat mold was used as control (without microstructure). The preparation of the films was made using non-mammalian components as described by Acevedo et al. [2] with minimal modifications. This formulation was based on a composite preparation by combining four components with well-defined properties related to bioactivity (salmon gelatin), crosslinking (calcium alginate), gelling (agarose), and plasticizing (glycerol). Salmon gelatin is a functional protein containing RGD-sequences allowing cell adhesion and proliferation [12,13,14]. Briefly, a solution composed by salmon gelatin 1.2% (obtained as described by Díaz et al. [23]), sodium alginate 1.2% *w/v* (Loba Chemie Pvt. Ltd., Mumbai, India), agarose 0.2% *w/v* (Loba Chemie Pvt. Ltd., Mumbai, India), and glycerol 1.0% (Merck, Darmstadt, Germany) was prepared at 50 °C with gentle agitation for 4 h. The solution was poured into the mold, regulating the volume in order to obtain 4 mm of height. The solutions were kept for 3 days at 10 °C to allow water evaporation, obtaining low moisture content films.

Prior to cell seeding, the films were soaked in CaCl_2_ solution (70 mM) for 1 h to crosslink the alginate fraction and obtain a non-water-soluble material [8,24].

### 2.3. Cell Culture

The myoblast cell line C2C12 was used as a model of mammal muscle cells. This cell line has been used to test edible films and scaffolds for in vitro meat [2,8]. The cell line was purchased from the European Collection of Cell Cultures (ECACC) and supplied by Sigma-Aldrich (St. Louis, MO, USA). The cells were cultured and maintained under standard conditions for cell culture (37 °C and 5% CO_2_) using a standard proliferation medium as Dulbecco’s Modified Eagle Medium (DMEM) high glucose (Gibco, Life Technologies, Grand Island, NY, USA) with 10% fetal bovine serum (Biologicals Industries, Kibbutz Beit-Haemek, Israel), l-glutamine (2 mM), and antibiotics (100 U/mL of penicillin and 100 µg/mL of streptomycin) (Gibco, Life Technologies, Grand Island, NY, USA).

### 2.4. Cell Proliferation

To study the cell proliferation and to select an optimum cell density to load the films, the myoblasts were seeded at three concentrations in the range used to seed C2C12 on the scaffolds [2,10,15,25]: 1.5 × 10^5^, 2.0 × 10^5^, and 2.5 × 10^5^ cells/cm^2^. Then, they were incubated in a 12-well culture plate for 72 h with 800 µL of the standard proliferation medium, as described in Section 2.3. The cell cultures were sampled at 3, 24, 48, and 72 h after loading. The morphology of the cells during proliferation were analyzed at each time using epifluorescence microscopy as described below.

### 2.5. Microscopy

Three microscopy techniques were used with the protocols reported by Acevedo et al. [2] for the study of muscle cells cultured on films.

Optical stereo microscopy (Leica Microsystems, model EZ4HD, Heerbrugg, Switzerland) was used to characterize the surface of the microstructured molds.

Scanning electron microscopy (SEM) was used to characterize the films, with a Carl Zeiss SEM (EVO MA 10, Oberkochen, Germany). Prior the analysis, samples were coated with gold by using a diode magnetron sputtering (SPI Sputter Coater model 12161, West Chester, PA, USA).

Epifluorescence microspcopy (Nikon, Eclipse TS2FL, Tokyo, Japan) was used to study the morphology and spreading of the cells cultured onto the films. Rhodamine-phalloidin and Hoechst 33342 (Invitrogen, Thermo Fisher Scientific, Eugene, OR, USA) were used to stain polymerized actin and nucleus, respectively.

### 2.6. Spreading Assay

The spreading of the cells cultured onto the microstructured films was assessed by means of image analysis using epifluorescence microscopy and ImageJ software v.1.52 (NIH, Bethesda, MD, USA). The area of the cells in each image was calculated as the size of the red stain emitted for rhodamine-phalloidin staining [26]. At least 10 images were analyzed per sample. Spreading was calculated as the ratio of cell area per image area and it was informed at different times.

### 2.7. Myogenic Differentation

Myoblasts were seeded onto the films at 2.5 × 10^5^ cells/cm^2^. Then, they were incubated in 12-well culture plate for 72 h with 800 µL of standard proliferation medium, as described previously. After proliferation phase (72 h), the proliferation medium was changed by differentiation medium (DMEM high glucose with 2% horse serum, 2 mM l-glutamine and antibiotics). Horse serum was purchased from Gibco (Life Technologies, Auckland, New Zealand). The day at which medium was changed it was called day zero of differentiation.

The differentiation phase was studied for 2 weeks. Every 2 days, the cells were gently washed, and the medium was replaced. The sampling to quantify gene expression (desmin, myogenin, and myosin heavy chain) using real-time quantitative polymerase chain reaction (RT-qPCR) was performed at 0, 7, and 14 days of the differentiation phase, as described below.

### 2.8. Gene Expression

The gene expressions of desmin, myogenin, and conventional myosin heavy chain class II were quantified using RT-qPCR. Desmin, myogenin, and myosin heavy chain are biomarkers of early, intermediate, and late myogenic differentiation, respectively [21].

The primers were designed using the software Amplifix 1.5.4 (INP, Marseille, France) and PrimerBlast (NCBI, Bethesda, MD, USA). Primer sequences used are shown in Table 1. The primers eukaryotic translation elongation factor 2 (Eef2) and porphobilinogen deaminase (PBGD) were designed to be used as alternatives of housekeeping genes.

Total RNA was isolated using commercial kits (TRIzol, Invitrogen, Carlsbad, CA, USA; RNeasy Plant Mini Kit, Qiagen, Hilden, Germany) according to the manufacturer’s instructions. Nanodrop One equipment (Thermo Fisher Scientific, Wilmington, DE, USA) was used to quantify concentration and quality ratios of RNA. Standard 1% agarose gel was used to check RNA integrity. Additionally, total DNA was quantified using Nanodrop previously extracted by using EZNA Tissue DNA kit (Omega Bio-tek, Norcross, GA, USA).

The commercial kit TURBO DNA-free (Invitrogen, Thermo Fisher Scientific, Vilnius, Lithuania) was used to treat previously the samples to remove DNA traces from the RNA extraction. Synthesis of cDNA (complementary DNA) was carried out with a commercial kit Improm-II (Promega, Madison, WI, USA) using the following components contained in the kit: dNTPs (0.5 mM), random primers (25 µg/mL), and reverse transcriptase (8 U/µL).

Control reactions without reverse transcriptase (-RT) were performed to screen for genomic DNA contaminations. The temperature profile of the cDNA synthesis protocol was as follows: 5 min at 70 °C, 5 min at 4 °C, 5 min at 25 °C (annealing), 60 min at 42 °C (synthesis), and 15 min at 70 °C (inactivation).

The RT-qPCR reaction was performed in the system AriaMx Real Time PCR (Agilent, Santa Clara, CA, USA) using Master Mix Brilliant II (Agilent, Santa Clara, CA, USA), primers (0.25 µM), double-distilled water, 2 µL cDNA, and 2 µL of diluted sample (1:2). The temperature profile was as follows: 10 min at 95 °C, 40 cycles (15 s at 95 °C, 15 s at 60 °C, 15 s at 72 °C), and followed by a melt curve analysis.

Relative gene expressions were evaluated using the Pfaffl method [27]. Results were normalized using as control cells cultured without film on commercial flasks (12-well plates) by 72 h, corresponding to the end of the proliferative stage or day zero of differentiation. The housekeeping gene used was PBGD, which was selected to show low variation. Values of CT (threshold cycle) for PBGD and Eef2 are shown in Table 2 for all conditions studied.

### 2.9. Statistics

Statistical significance was determined by Student’s *t*-test, ANOVA, or Tukey’s test. Differences were considered to be significant when *p* < 0.05. All data obtained are expressed as mean ± standard deviation.

## 3. Results and Discussion

### 3.1. Micromolding

The fabrication of dense skeletal muscle tissue requires a uniform cell alignment and reproducible microarchitecture. Additionally, orienting the fibers towards one direction could be beneficial in in vitro meat development, as it resembles the muscle native structure [4]. Then, a suitable scaffold must promote the alignment of the progenitor muscle cells.

It is well known that myoblasts (muscle progenitor cells) need to be aligned to correctly develop the myogenic differentiation, and later to form muscle fibers. In our case, the fabricated microstructured mold featured oriented parallel microchannels (see Figure 1B,C), where the myoblasts could proliferate, followed by differentiation to myocytes and finally fusing to fibers expressing myosin heavy chain.

**Figure 2:**
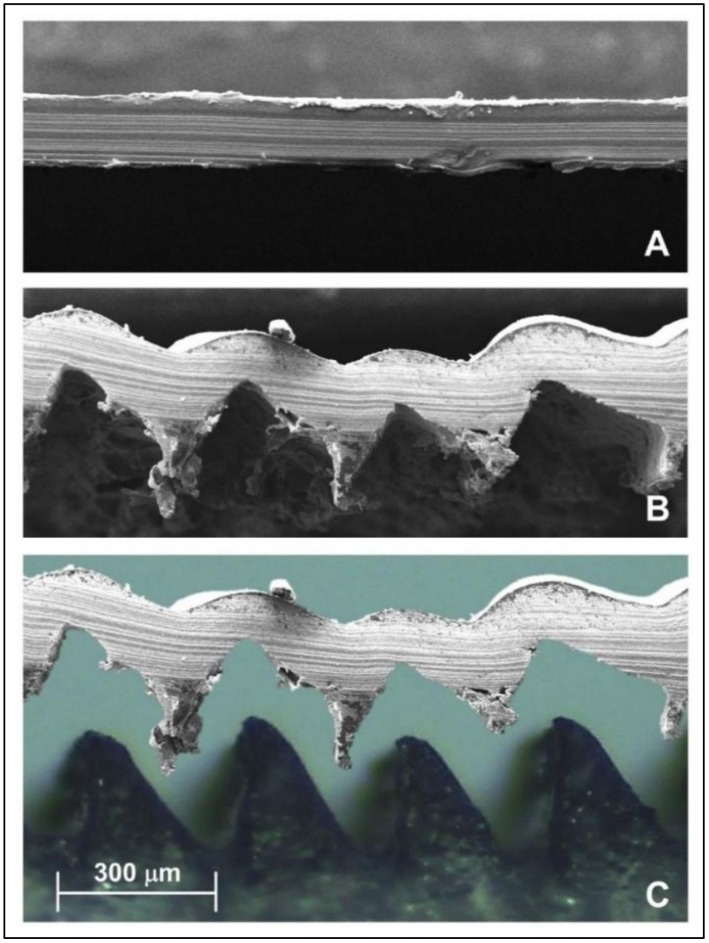
Microscopy of the films (scale bar 300 µm). (**A**) Scanning electron microscopy (SEM) image of the flat film. (**B**) SEM image of the microstructured film. (**C**) Photomontage between an optical image of the microstructured mold and a SEM image of the microstructured film.

Figure 2 shows the films formed after the solvent-casting. The flat mold produced a flat film (Figure 2A). On the other hand, the micromolding produced a film with the expected microchannel geometry (Figure 2B).

Figure 2C is a photomontage between a selected section of microstructured mold and microstructured film, using scaled microscopy images. The micromolding and peeling process of the film onto the mold can be schematically observed. Each ridge of the mold produced a channel in the film with similar shape and dimension.

### 3.2. Proliferation of Muscle Fiber-like Biomass

**Figure 3:**
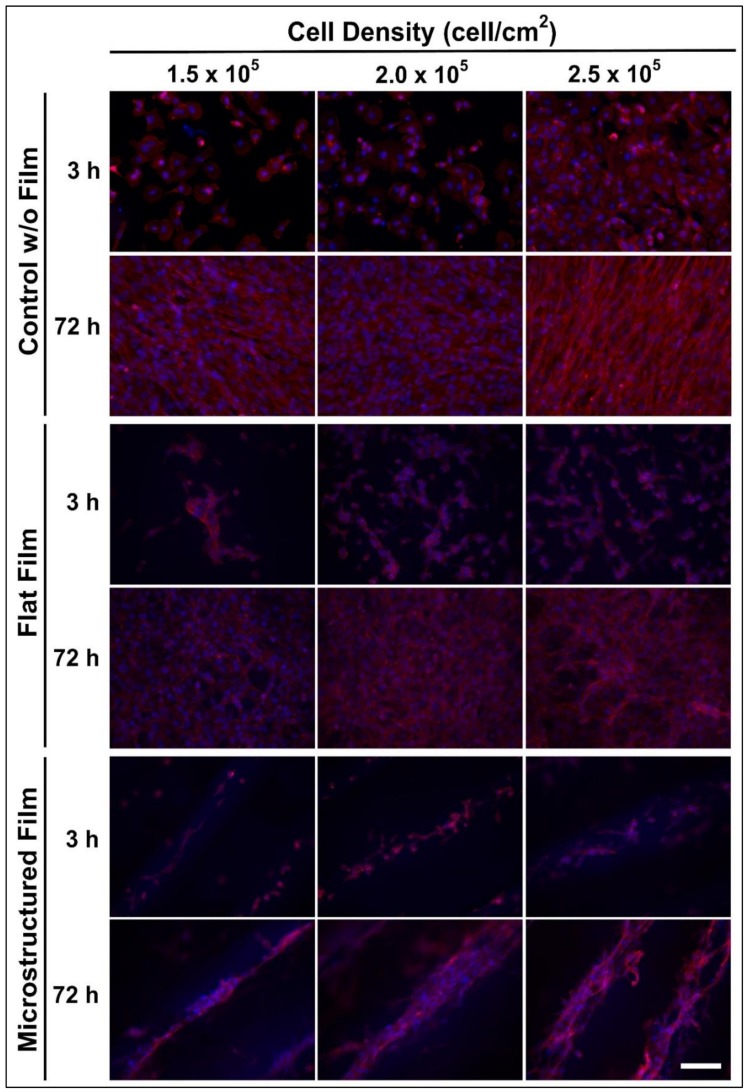
Epifluorescence microscopy of cells cultured on flat and microstructured films. Red color shows actin with rhodamine-phalloidin staining. Blue color shows cell nuclei with Hoechst staining. Scale bar 100 µm.

The first stage to produce in vitro meat is related to cell proliferation to obtain a high number of cells, which is necessary to accomplish the fusion and to form fibers in later stages. Figure 3 shows muscle cells adhered on the films after 3 h of seeding, at three initial densities, and in their proliferation phase after 72 h.

The cells cultured without film (control) showed the known morphology of C2C12 myoblasts. It is well known that in the undifferentiated condition, the C2C12 myoblasts are flat, fusiform, or star-shaped mono-nucleated cells, and they express actin and focal contacts [28]. Cells cultured on the control surface proliferated by 3 days, exhibiting the typical behavior of confluent myoblasts, showing intense growth, actin expression (red staining), and some signs of cell fusing with orientation, being more evident for the highest concentration used.

Morphology of the cell culture on the films were different when compared against the control (cells cultured without film). However, it has been reported that morphology of C2C12 depends of the kind of surface over which it is cultured, for instance, fibroblastic shape on plastic, rounded on Matrigel [10], or even star-shaped when cultured on nanocoated surfaces [29]. The morphology of myoblasts, myocytes, and myotubes in scaffolds can vary substantially from those in monolayer culture [2,8,10]. Therefore, the microscope observations shown in Figure 3 agree with those of C2C12 cultured in different surfaces.

The cells cultured on the flat films grew unorganized for all the densities studied. The literature indicates that skeletal precursor muscle cells can be cultivated and differentiated in vitro, but they can invariably proliferate and differentiate to unorganized and nonfunctional myotubes [18]. Although these results demonstrate that the formulated material is biocompatible, non-toxic, and allows myoblast proliferation, the film with a flat surface structure cannot be regarded as functional because it did not induce any cell orientation. On the contrary, the cells seeded on the microstructured film grew parallel and aligned to the constructed channels, forming fiber-like structures with a diameter in the range of 70 to 90 µm, which were similar in dimension to meat fiber diameters for beef (≈70 µm) and pork (≈90 µm) [22].

**Figure 4:**
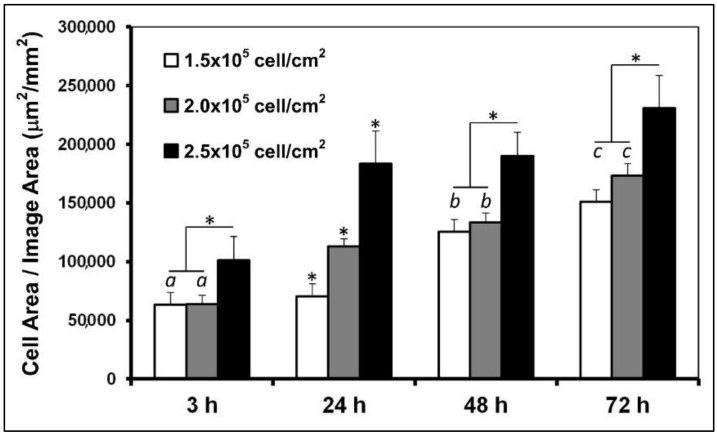
Spreading of muscle cells cultured on microstructured films. Asterisk (*) shows significant difference (*p* < 0.05; ANOVA; *n* = 10). Bars with same letter indicate no significant difference between them (*p* > 0.05; Tukey’s test; *n* = 10).

Figure 4 shows the cell spreading (cell area increasing over time) onto the microstructured film during the proliferation phase. It is clear that cell spreading on the microchannels was dependent upon the initial cell density. The higher cell density used to load the microstructured films (2.5 × 10^5^ cell/cm^2^) allowed us to obtain the best spreading of the fiber-like biomass.

Important requirements for functional muscle cells are the parallel alignments of myofibrils with myosin and other proteins, which are needed for creating direct forces and functional use [30]. In particular, there has been a long-standing research interest to direct the fusion of muscle precursor cells (myoblasts) to generate myotubes, which can mature into myofibers [18]. The formation of an aligned fiber-like structure is evidence that the microchannels formed on the film are suitable for potential production of in vitro meat. The flat film produced non-aligned cell proliferation, which was not adequate for obtaining muscle fibers. Nevertheless, to confirm the appropriate differentiation to muscle fibers, a quantification of the expression of myogenic markers was necessary. For this, the cell density that we found most suitable was selected (2.5 × 10^5^ cell/cm^2^) to study the myogenic markers at the differentiation phase.

### 3.3. Expression of Genes of Myogenic Differentiation

After cell seeding on the films (2.5 × 10^5^ cell/cm^2^), they were cultured with the proliferation medium for 72 h. Then, it was changed to differentiation medium to start the differentiation phase.

**Figure 5:**
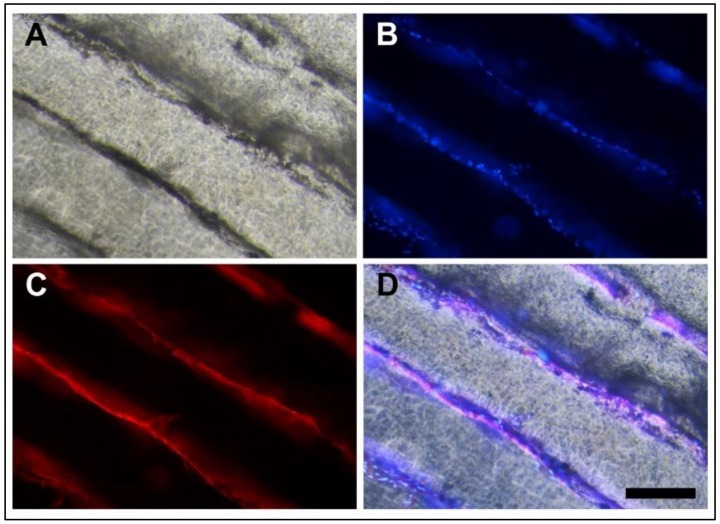
Optical/epifluorescence microscopy of fibers on microchannels after medium change (scale bar 200 µm). (**A**) Optical microscopy image. (**B**) Epifluorescence microscopy using Hoechst staining (blue shows cell nuclei). (**C**) Epifluorescence microscopy using rhodamine-phalloidin staining (red shows actin). (**D**) Merge of the microscopy images.

Figure 5 depicts an optical/epifluorescence microscopy analysis after the medium change, which shows a muscle fiber morphology growing onto the parallel microchannels of the film. This image indicates that the microchannel acted as a guide for the parallel alignment of cells, because the cell orientation had the same shape of the channels, which was still maintained after medium change. 

On the other hand, in order to relatively compare the initial biomass starting the differentiation phase between films, a total DNA quantification was performed. The cell concentration seeded on both films for proliferation was the same, and the number of cells at the differentiation phase was checked indirectly. Similar values of total DNA in both films were measured, being close to 1.5 µg/cm^2^ (1.55, 1.58, and 3.08 µg/cm^2^ in microstructured, flat, and control without films, respectively). Then, equivalent number of cells were differentiated in both films. However, in the microstructured film, the cells were concentrated onto the channels, increasing the cell density locally.

The effects produced by the channel microstructure on cells, local increase of cell density, and parallel alignment of cell elongation could have been triggering the development of a dense and aligned muscle structure onto the microchannel, likely followed by the expression of early myogenic biomarkers, and then the myosin transcription. This concept was studied using RT-qPCR.

**Figure 6:**
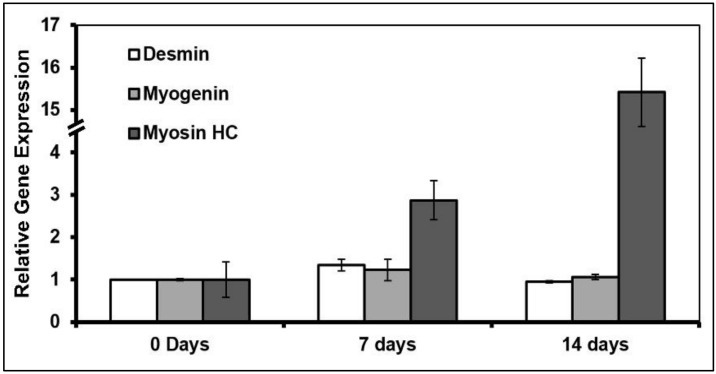
Gene expression of myogenic markers of cells cultured without films (commercial flasks).

Samples for RT-qPCR quantification of myogenic biomarkers were taken at 0, 7 and 14 days of starting differentiation, and the values were normalized using cells cultured without film (on commercial flasks) at day zero of differentiation to relativize to a single and known control. Gene expression of cell differentiation without films at 0, 7, and 14 days are shown in Figure 6. The cells maintained the expression of desmin and myogenin in the first week, but they expressed a high level of myosin heavy chain at day 14, indicating advanced myogenic differentiation after 2 weeks.

**Figure 7:**
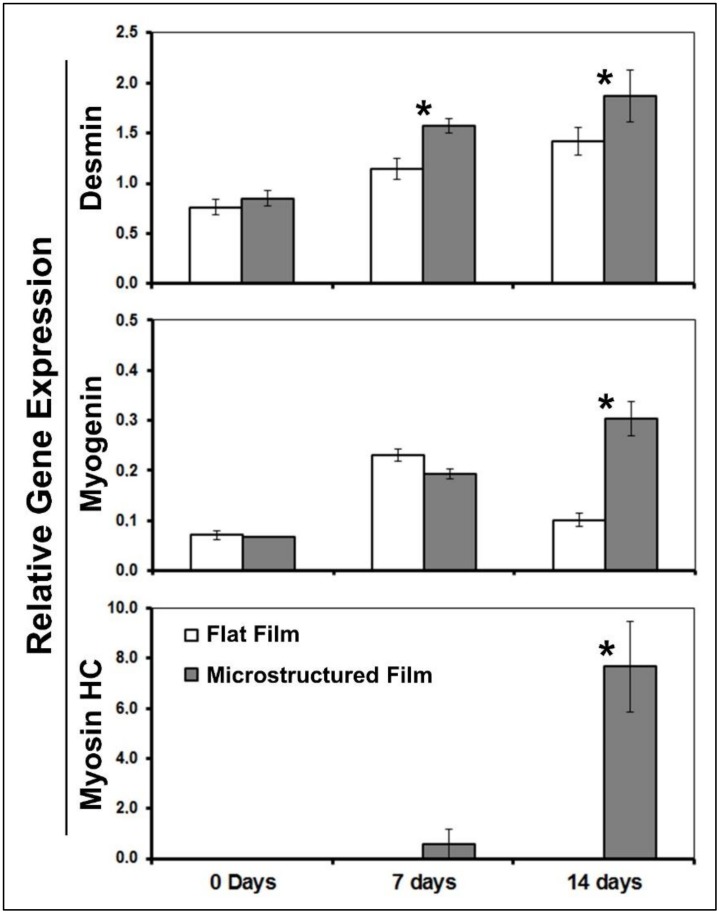
Gene expression of myogenic markers of cells cultured on flat and microstructured films. Asterisk (*) shows significant difference (*p* < 0.05; *t*-test; *n* = 3) between flat and microstrucured film.

Figure 7 shows the expression of desmin, myogenin, and myosin heavy chain in the flat film and microstructured film. At day zero, the expression of desmin and myosin by the cells cultured on flat and micrustructured films did not show significant differences (*p* > 0.05; *t*-test). Cells on both film types did not express myosin heavy chain at day zero, indicating the typical behavior of non-differentiated myoblasts [21,31].

After 7 days in contact with the differentiation medium, the expression of desmin by cells in the structured film was higher than flat film (*p* < 0.05; *t*-test). Desmin is one of the earliest known myogenic markers for skeletal muscle [32], and the increase in gene expression indicates that the microchannels molded onto the film can promote the differentiation to muscle cells.

The expressions of myogenin by cells at 0 and 7 days of differentiation did not show significant differences when they were cultured on flat and microstructured films (*p* > 0.05; *t*-test). The myogenin is an intermediate biomarker of the myogenic differentiation [21]. Seven days or less is considered an early state of differentiation; therefore, in finding no differences in gene expression, it cannot be concluded that microchannels increased the formation from myoblasts to myocytes before 1 week. Myogenin is a marker for the entry of myoblasts to the differentiation pathway, and the expression only occurred in myocytes [31]. At day 14, the expression of myogenin was significantly higher (*p* < 0.05; *t*-test) in the microstructured film. This indicated that microchannels increased the formation of myocytes between 7 and 14 days, which would be considered a signal of the differentiation. The relative expressions of myogenin in all conditions evaluated were less than 1 (control cells cultured without film on commercial flasks), indicating that the non-edible plastic surface is more suitable for myogenin transcription than the edible films. However, although the expression was less than the control, it was effectively detected in all the conditions evaluated, indicating a progression of intermediate myogenesis.

The expressions of myosin heavy chain by cells cultured on both films were not detectable at day zero. However, after 2 weeks, the expression in the microstructured film markedly increased and was much higher than the gene expression by cells on the flat film (*p* < 0.05; *t*-test). As stated in other section of this manuscript, the myosin heavy chain is a well-known marker of the muscle differentiation [19]. Its gene expression currently offers the most suitable marker of muscle fiber composition and generally coincides with a well-defined set of contractile and metabolic properties [20].

Gene expression of desmin, myogenin, and myosin heavy chain demonstrated that the parallel arrange of microchannels designed onto the edible film improved the myogenic differentiation. These data and the formation of a fiber-like morphology, as is shown above, are results of obtaining a muscular structure that can be used in further experiments with bovine satellite cells in the context of in vitro meat production.

## 4. Conclusions

The development of a microstructured template allowed the fabrication of films with adequate geometry that promoted parallel alignment of myoblasts seeded onto them, which is an essential requirement for further formation of muscle fibers.

The fiber-like spreading on the surface of the microchannels was dependent of the cell density previously loaded onto the films. High cell densities facilitated acceptable and fast fiber spreading. This feature is a desirable condition aiming at scaled-up processes where bioreactors are used for rapid biomass generation.

The formation of a muscle fiber-like structure in addition to the expression of myosin heavy chain strongly suggest the obtention of muscular tissue structure. Hence, the design of microstructured film could potentially be used as a tool for the production of in vitro meat. However, further experiments using bioreactors and primary bovine cells are necessary to translate the results from a myoblast cell line cultured in flasks to an economically viable production system.

## Figures and Tables

**Table 1:** Genes and primer sequences used in real-time quantitative polymerase chain reaction (RT-qPCR).

Gene	Name	Accesion GenBank	Primer Sequences	Amplicon Length
Des	Desmin	NM_010043.2	Fw: 5’-GATGCAGCCACTCTAGCTCGTATT-3’ Rv: 5’-TTCTTAGCCGCGATGGTCTCATAC-3’	218 bp
Myog	Myogenin	NM_031189.2	Fw: 5’-AGAAGCGCAGGCTCAAGAAAGT-3’ Rv: 5’-AGTTGCATTCACTGGGCACCAT-3’	222 bp
Myh2	Myosin heavy chain	NM_001039545.2	Fw: 5’-CCTCTTATTTCCCAGCTGCACCTT-3’ Rv: 5’-GTCACTTTCCCTGCATCTTTGCTC-3’	242 bp
PBGD	Porphobilinogen deaminase	NM_001110251.1	Fw: 5’-TGGCGATGCTGAAAGCCTTGTA-3’ Rv: 5’-GTTTTCCCGTTTGCAGATGGCT-3’	239 bp
Eef2	Eukaryotic translation elongation factor 2	NM_007907.2	Fw: 5’-ATCGTGGAGAACGTCAACGTCA-3’ Rv: 5’-TGCCATTGGCCGGATCAAAGTA-3’	274 bp

**Table 2:** Threshold cycle (CT) values obtained in different conditions to select the housekeeping gene.

Gene	Surface	Days of Differentiation			ΔCT (Max-Min)	Coefficient of Variation
		0	7	14		
PBGD	Commercial Flask	25.5 (±0.08)	25.3 (±0.06)	25.2 (±0.01)	0.3	0.007
	Flat Film	26.8 (±0.29)	27.0 (±0.11)	27.4 (±0.00)	0.6	0.011
	Microstructured film	27.2 (±0.11)	26.6 (±0.15)	27.2 (±0.30)	0.6	0.013
Eef2	Commercial Flask	20.7 (±0.06)	19.9 (±0.12)	20.2 (±0.37)	0.8	0.020
	Flat Film	21.8 (±0.30)	21.8 (±0.01)	22.4 (±1.78)	0.6	0.016
	Microstructured film	22.6 (±0.02)	20.9 (±0.13)	21.2 (±0.01)	1.7	0.042
